# A Systematic Review of Botulinum Toxin Injection in Pediatric Dystonia

**DOI:** 10.3390/toxins16070289

**Published:** 2024-06-26

**Authors:** Andrea Rasera, Giovanna Maddalena Squintani, Maria Angela Cerruto

**Affiliations:** 1 Neurology Unit, “Azienda Provinciale per i Servizi Sanitari”, Trento “Santa Chiara” Hospital, Largo Medaglie d’oro 9, 38122 Trento, Italy; 2 Section of Neurophysiology, Neurology Unit, “Azienda Ospedaliera Universitaria Integrata Verona”, Verona “Borgo Trento” Hospital, Piazzale Aristide Stefani 1, 37126 Verona, Italy; giovannamaddalena.squintani@aovr.veneto.it; 3 Urology Clinic, Department of Surgery, Dentistry, Paediatrics and Gynaecology, University of Verona, Piazzale Aristide Stefani 1, 37126 Verona, Italy; mariaangela.cerruto@univr.it

**Keywords:** botulinum toxin, pediatric dystonia, dystonia treatment

## Abstract

Botulinum toxin (BT), a first-line treatment for focal dystonias in adults, has gained USA Food and Drug Administration approval for pediatric upper and lower extremity spasticity and sialorrhea, though its use in children younger than 2 years old is still considered off-label treatment for all pathologies. Dosing, treatment strategies and outcome measures lack international consensus, and they are often extrapolated from adult or spasticity guidelines. This review aims to evaluate the best available evidence on the efficacy and safety of BT therapy in pediatric dystonia (age under 21 years old), isolated or associated with other medical conditions. A comprehensive search in PubMed, Scopus and Web of Science was conducted, including only articles in English. Although no randomized controlled trials are still present, 12 articles were included with an overall of 57 patients. All the papers demonstrate that BT can improve motor function, decrease pain and ameliorate quality of life, with minimal adverse effects in pediatric patients affected by pure or mixed dystonic motor disorders. Despite the low level of evidence, our review shows that BT could be an efficacious treatment for these pediatric patients. The frequent generalized involvement, together with the heterogeneous nature of childhood dystonic forms, sometimes intermingled with spasticity, prompts further multicenter clinical trials or prospective studies with a higher level of evidence to shed light on the efficacy and safety profile of BT in pediatric dystonia.

## 1. Introduction

Dystonia refers to sustained or intermittent muscle contractions with abnormal posture movements that are usually repetitive and patterned [1]. This recent definition of dystonia applies to both adults and children and is similar to that published by the Taskforce on Childhood Movement Disorders [2]. In children, dystonia is generally more often generalized than the adult-onset forms. According to the latest classification, dystonia is classified along two axes of clinical characteristics and etiology. Age at onset is one of the four subclasses on Axis I and is divided into five subcategories: infancy (birth-2 years), childhood (3–12 years), adolescence (13–20 years), early adulthood (21–40 years) and late adulthood (>40 years). Body distribution is the second subclass on Axis I and divides dystonia into focal, segmental, multifocal, generalized or unilateral types [1]. There are various different causes of dystonia in children, which make diagnostic workup often challenging because of the heterogeneity of clinical presentation. The onset of symptoms and signs during growth development or at a given age makes diagnosis and treatment management especially challenging for neurologists [3].

Among the inherited forms, dystonia is “isolated” in some cases, “combined” when associated with other movement disorders and “complex” in other complex diseases or neurological manifestations, such as seizures or intellectual disability [4]. Early onset dystonia (DYT1) is the most common isolated form of inherited dystonia, while dopa-responsive dystonia presenting in childhood and adolescence is the most frequent combined form. Complex inherited dystonia encompasses a broad spectrum of hereditary neurodegenerative and metabolic disorders, including homocystinuria, phenylketonuria, neuroacanthocytosis, Wilson’s disease, mitochondrial disorders and gangliosidosis. Finally, autoimmune, post-traumatic, hypoxic–ischemic, drug-induced, infective, neoplastic, vascular or psychogenic forms are the most frequent types of acquired dystonia.

Complex clinical pictures are common in pediatric motor disorders; cerebral palsy (CP), in particular, is categorized into spastic, dyskinetic and ataxic forms [5]. The term “dyskinetic cerebral palsy” is referred to as the clinical presentation of CP characterized by abnormal postures or movements that resembles dystonia or/and choreoathetosis [6]. Sometimes clinical phenotypes are not always pure and may coexist: dystonic postures may also be difficult to distinguish from spasticity, particularly those affecting the lower extremities and when they occur in the same limb [7,8].

The current treatment options for childhood dystonia are physical and supportive therapy, oral medications, botulinum toxin (BT) injection and neurosurgical procedures [4,9]. BT binds to specific sites on the presynaptic cholinergic nerve terminal, thus inhibiting the release of acetylcholine into the synaptic cleft that results in neuromuscular blockade [10]. The effect of a BT injection typically appears within 1 week and peaks at around 4–6 weeks, before gradually disappearing at 2.5–3 months [10]. An incorrectly performed infiltration or an excessive dose of BT in children may cause local or systemic adverse effects, such as dysphagia, gastrointestinal paresis and respiratory muscle failure [11]. BT treatment is considered the preferred option for treating most types of focal dystonia [9], but its use in children is limited mainly owing to the prevalence of generalized dystonic forms [12]. As reported for adults, BT injection in children may be a therapeutic option as relief treatment for most not-well-controlled symptoms of segmental or generalized dystonia when alternative treatments have proven suboptimal [9]. However, there is limited evidence for its role in the treatment of dystonia in children; in particular, there are no specific reviews because there is usually no clear identification and description of dystonic features in cases of muscular hyperactivity involving the pediatric population. Regarding the outcome measures, they are not standardized according to the most updated literature, and they are mostly based on clinical evaluation, motor function scales (range of motion—ROM; modified Ashworth scale—MAS) or dystonia scales (Burke–Fahn–Marsden dystonia scale—BFMDS; Barry–Albright dystonia scale—BADS).

The aim of this review is to evaluate the efficacy and safety of BT treatment in pediatric dystonia. To better achieve this objective, we conducted a systematic review of the literature on the best available evidence and thoughtful recommendations.

## 2. Results

**Figure 1:**
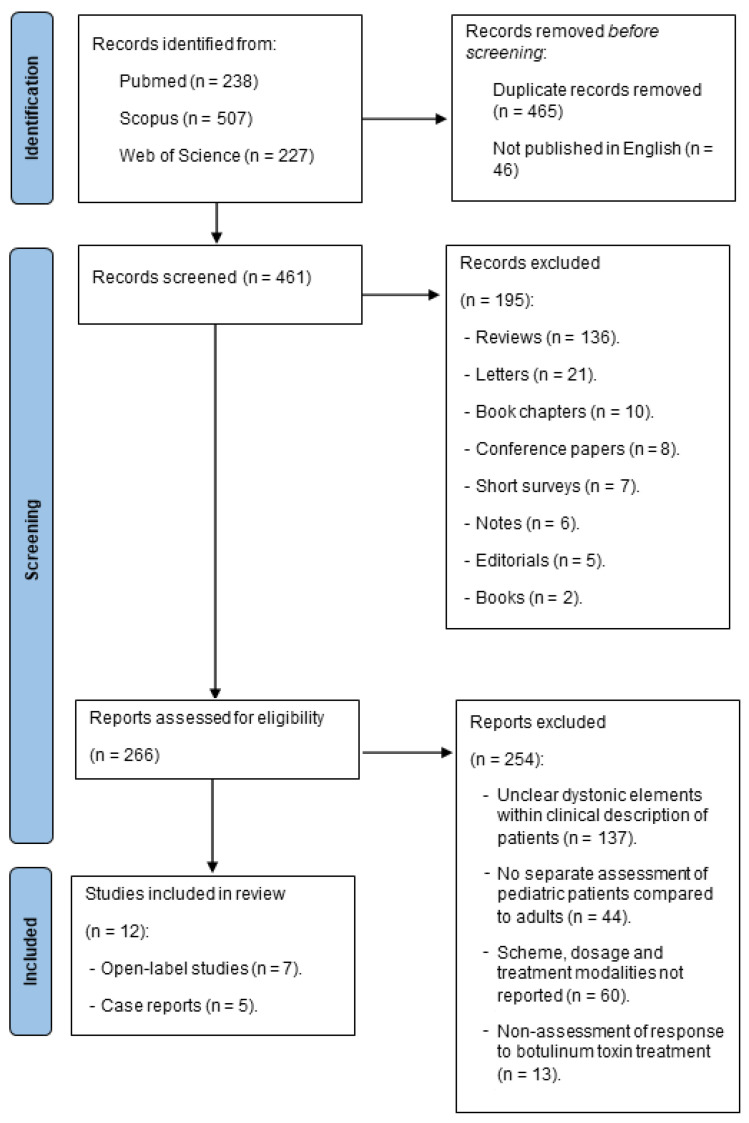
PRISMA flow diagram for systematic review.

A total of 461 records were evaluated for screening and 266 of them were assessed for eligibility. Finally, only 12 manuscripts were included for this review: 7 open-label trials and 5 case reports. We found no Class I or II studies on the use of BT in pediatric dystonia. The Preferred Reporting Items for Systematic Review and Meta-Analyses (PRISMA) diagram (Figure 1) shows the literature research results. Table 1 presents a summary of our findings. Four open-label studies reported outcomes after treatment for dystonia associated with CP, the so-called dyskinetic cerebral palsy (DCP) [13,14,15,16], while others focused on focal dystonia [17,18] or opisthotonos [19]. Three case reports reported outcome measures of patients with a defined genetic condition associated with generalized dystonia: pantothenate kinase-associated neurodegeneration (PKAN) [20,21] and Hallervorden–Spatz syndrome (HSS) [22]. Another case report described lower limb congenital dystonia associated with thoracolumbar myelomeningocele [23]; the etiology of generalized dystonia described in another report remained undetermined [24].

Overall, 57 patients were included in this review: they were identified within larger cohorts, which included patients with other non-dystonic muscle hyperactivity; patients suitable for inclusion ranged from 5 to 16 per article.

Although the review process identified only a few articles, patient age was highly heterogeneous, ranging from 6 months to 20 years old, and even maintained within the individual cohort studies.

Most studies reported on patients treated with Onabotulinum toxin type A (Ona BTA) (Botox) [13,16,19,21,24] or Abobotulinum toxin type A (Abo BTA) (Dysport) [17,20,22,23] or both [15], while two studies reported injection of Rimabotulinum toxin type B (Rima BTB) (Myobloc) [14,18]. The BT dose ranged from 4 to 23.8 U/kg for Ona BTA, from 4.8 to 30 U/kg for Abo BTA and from 50 to 200 U/kg for Rima BTB. Regarding the injection technique, anatomical markers were used by four authors [13,17,19,24], while ultrasound-assisted injections were used by two researchers [15,18]; two authors used electromyographic muscles identification [14,23], whilst in the other four cases the injection method was not specified [16,20,21,22].

For the reporting of treatment outcomes, some studies evaluated motor function recovery based on quantitative motor scales [13,14,16,17,18,21] or on empirical clinical improvement [19,20,22,23,24].

Three studies also analyzed quality of life [14,20,21]. One study outcome was improvement in the pain profile [15]. All the studies reported completely or partially achieved their outcome, demonstrating that BT treatment had a positive effect on children with dystonia. Furthermore, BT injection was found to be devoid of side effects and therefore highly safe. Three open-label studies reported sporadic transient post-injection weakness in a few patients [13,14,19].

The evidence level was IV for all the articles as well as the recommendation class (U), showing the insufficiency of the data to either endorse or discourage the use of BT as a treatment.

## 3. Discussion

The present review revealed a lack of strong recommendation for the use of BT in pediatric dystonia, with the articles proving there is a low level of evidence (IV) and insufficient data to support or refute the use of BT as a treatment (class U). This conclusion is mainly addressed by the fact that we found no RCTs and only studies with small study populations. The reasons are three-fold: first, focal and segmental dystonia, which could benefit most from BT therapy, are less frequent than generalized forms in children [11,12]; second, BT is often administered after pharmacological or conservative therapy has failed or as a second-line therapeutic option in pediatric dystonia [9,17]; and third, the young age of patients often precludes the approval of RCTs by ethics committees [25].

The strongest evidence we found concerns dystonia associated with cerebral palsy. DCP can be found in 6–15% of patients with CP [4] and is characterized by typical dystonic features, such as the overflow phenomenon, pattern change with movements, postures or sensory stimulations, and is occasionally associated with fixed postures, hyperreflexia or “catches” [8].

While a dystonic pattern within a syndrome characterized by spasticity may not always be easy to recognize [2,8], it can be decisive in the therapeutic approach to the patient. A recent study highlighted that recognizing dystonia within CP can impact diagnostic and therapeutic workup and that the presence of concomitant dystonia is a positive predictor of response to BT treatment [26].

Several studies reported clinical progress in global function [13], while others reported increased ability on arm-reaching tasks [14], with no serious side effects (only transient weakness) in patients with CP-associated dystonia. Valentine and co-workers [16] reported motor improvement in CP patients with mixed dystonic and spastic phenotypes, although no clinical distinction was made between patients with pure spastic forms and those with associated dystonia.

Other studies based self-reported beneficial outcomes after BT treatment on the quality of life and pain profile questionnaires [14,15]. The analgesic effect is probably due to the reduction in muscular tone and release of excessive muscular tone on nerves and vessels. There is also a direct analgesic effect of BT treatment on the pain transmission system via the modulation of the biological effect of inflammatory substances and other pain mediators [27]. The latter evidence may have a key role in a future perspective on the management of CP patients, as pain symptoms are present in 32% to 74% of patients [28] and the concomitant presence of dystonia in CP could be evaluated as a positive predictor of BT therapy on non-motor symptoms. The case reports also highlighted the therapeutic potential of BT in pediatric dystonia, with improvement in the clinical symptoms, quality of life and social relationships by reducing disabling hypertonic muscular activity in specific sites [20,24].

Although the level of evidence and recommendation grade were low (IV and U, respectively), the articles included in this review were all unanimous in highlighting the efficacy and safety of BT treatment in pediatric dystonias. To date, they represent the best available evidence and, although limited, they may support the use of BT in pediatric patients affected by dystonia.

RCTs should be warranted in future studies to evaluate the efficacy, tolerability and safety of BT, but it will be necessary to overcome some limitations mainly related to the stringent approvals in the datasheet of commercially available BT and to the lack of standardization of dosing in the pediatric population.

Although BT is considered a first-line treatment for most types of focal dystonia in adults [29], there is limited approval for its use in this condition in the pediatric population. Ona BTA and Abo BTA are now US FDA-approved for upper and lower extremity spasticity in children (>2 years); Inco BTA is approved for pediatric sialorrhea and pediatric upper extremity spasticity (>2 years), while BT injection is considered off-label treatment in children under 2 years.

BT dosing is based on the patient’s body weight and the size and number of muscles to be injected. At present, international guidance for BT dosing in pediatric dystonia is lacking. Injection doses are often extrapolated from adult guidelines or from guidelines for treating spasticity in childhood. Dose estimation is generally based on the total units given during a single treatment session; total units/kg body weight per session; and units per muscle, units/injection site or units/kg of body weight/muscle. The 2009 European Consensus on the Use of BT for Children with Cerebral Palsy recommended a maximum dose of 400 U (or 20 U/kg) of Ona BTA, 1000 U (or 20/30 U/kg) for Abo BTA and 5 U/kg for Inco BTA [30]. Dose adjustment takes account of the severity of the dystonia, other concomitant diagnoses such as spasticity, dysphagia, breath impairment, and activity of the injected muscle [31]. Because many muscles are involved in idiopathic torsion dystonia, BT treatment is usually impracticable [32].

The efficacy, tolerability and safety of BT in adult dystonias is well established for all types of dystonias [33], and all types of BT (Ona BTA, Abo BTA, Inco BTA and Rima BTB) have been approved by the FDA with a high level of recommendation (mainly class A and B), although with slight differences regarding their use for the specific types of dystonia [34]. The results of our review are encouraging but not consistent with the level of evidence obtained in adults in which several RCTs have been performed. Moreover, currently there are no systematic reviews of BT treatment for pediatric dystonia; the reviews on CP generally include patients with either spastic or dystonic forms combined [7] or mixed disorders (i.e., musculoskeletal disease) [35].

Regarding adult dystonias, the AAN guidelines [29] supporting BT utilization are obtained from data of several RCTs targeting populations of subjects with isolated and specific dystonic pathology, and the outcomes are established based on standardized, quantitative measures, such as the Tsui scale, 5 Toronto Western Spasmodic Torticollis Rating Scale, Cervical Dystonia Severity Scale and Jankovic Rating Score. The main issue that should be addressed in near-future RCTs on pediatric dystonias is the possibility to perform studies on uniform patient populations (generalized and focal dystonia), and the prevalence of generalized forms in pediatric patients represents one of the most relevant limitations; additionally, the fact that pediatric population dystonia is rarely idiopathic and often associated with other muscular hyperactivity disorders and/or systemic diseases introduces another bias in the assessment of outcome measures.

Moreover, there are no dedicated rating scales to define outcomes for dystonias in the pediatric population. Future RCTs should identify defined and approved scales for children to quantify the efficacy of BT treatment, including also non-motor parameters, given that dystonia is not an isolated movement disorder but rather a multi-system disease [36] and BT has proved to have a “pleyotropic” effect, affecting also the central nervous system [37].

## 4. Conclusions

The treatment of pediatric dystonia is challenging, and BT therapy can play a decisive role; however, the evidence is still limited and the lack of RCTs does not provide for strong recommendations on its use in daily clinical practice. Research studies in this area are limited by two main factors:

- Childhood dystonia rarely occurs in idiopathic forms with focal or segmental involvement, limiting BT use in clinical practice.
- Rating scales to assess isolated or combined dystonia are lacking in the pediatric population, making outcome definitions and evaluations often arduous.

Our review provides evidence for the efficacy of botulinum toxin treatment on both motor and non-motor symptoms of pediatric dystonia, without major side effects. Nonetheless, the level of evidence is still low, and the strength of the recommendations is based mainly on expert opinion. Multicenter trials or larger prospective studies are therefore needed to better understand the efficacy, tolerability and safety profile of botulinum toxin in the treatment of pediatric dystonia.

## 5. Materials and Methods

For this systematic review, we followed the PRISMA statement [38]. We searched the scientific databases PubMed, Web of Science, Cochrane and SCOPUS for articles published in English in the last 40 years on BT injection for the treatment of pediatric dystonia (age < 21 years). The inclusion criteria were subjects under age 21 years and isolated dystonia or associated with other diseases. The search terms were “pediatric dystonia”, “childhood dystonia”, “adolescence dystonia”, “infant dystonia”, “young dystonia”, “treatment”, “botulinum”, “botulinum injection”, “young age dystonia”, and “botulinum toxin”.

Given the scarcity of randomized controlled trials (RCTs), non-randomized studies (i.e., open-label studies, retrospective studies, case series and case reports) were considered for review to gain a comprehensive overview of the currently best available evidence.

English-language titles and abstracts were reviewed in their full-text publication. Editorial commentaries, systematic reviews and meta-analyses were used to find additional articles. All the data extracted from the included manuscripts were recorded in an electronic database. All the outcomes regarding improvement in motor and/or non-motor symptoms were evaluated. All the records were retrieved independently by two neurologists (G.S. and A.R.); a third reviewer (M.A.C.) was recruited to reach a shared consensus when agreement could not be reached on the level of evidence.

All the records were reviewed completely and classified using the American Academy of Neurology (AAN) classification of evidence for therapeutic intervention and classification of recommendations [39,40].

AAN Classification of Evidence for Interventions [39]

- Class I. A randomized, controlled trial (RCT) with a masked or objective outcome assessment in a representative population. The relevant baseline characteristics are presented and substantially equivalent among treatment groups or there is appropriate statistical adjustment for differences. The following are required: (a) concealed allocation; (b) primary outcome(s) clearly defined; (c) exclusion/inclusion criteria clearly defined; and (d) adequate accounting for dropouts (with at least 80% of enrolled subjects completing the study) and crossovers with numbers sufficiently low to have minimal potential for bias.
- Class II. A prospective matched group cohort study in a representative population with a masked outcome assessment that meets criteria b–d above or an RCT in a representative population that lacks one criterion a–d.
- Class III. All the other controlled trials (including well-defined natural history controls or patients serving as own controls) in a representative population, where the outcome is independently assessed or independently derived by an objective outcome measurement.
- Class IV. Studies not meeting Class I, II or III criteria, including consensus, expert opinion or a case report.

AAN Classification of Recommendations (source American Academy of Neurology) [40].

A. Established as effective, ineffective or harmful (or established as useful/predictive or not useful/predictive) for the given condition in the specified population. (Level A rating requires at least two consistent Class I studies.)
B. Probably effective, ineffective or harmful (or probably useful/predictive or not useful/predictive) for the given condition in the specified population. (Level B rating requires at least one Class I study or two consistent Class II studies.)
C. Possibly effective, ineffective or harmful (or possibly useful/predictive or not useful/predictive) for the given condition in the specified population. (Level C rating requires at least one Class II study or two consistent Class III studies.)
U. Data inadequate or conflicting; given current knowledge, treatment (test and predictor) is unproven.

Strong levels of recommendation were based on the highest levels of evidence; when evidence from an RCT or a systematic review was lacking, practice-based evidence and expert opinion were suggested. This systematic review is not registered.

## Figures and Tables

**Table 1:** Characteristics of the studies included in this review.

Author	Age (Years)	Study Design (No. of Patients)	Disorder	Formulation, Total Dose	Injection Site	Outcome Assessment	Results	Adverse Events (%)	Level of Evidence	Recommendation
Seiff et al., 1989 [24]	16	Case report (*n* = 1)	Generalized and face dystonia	BTA, 80 U	OO	Clinical	Clinical improvement	None	IV	U
Heinen et al., 1995 [23]	6 months	Case report (*n* = 1)	Lower limb dystonia and myelomeningocele	Abo BTA, 10–12 U/kg (total dose 140 U in 2 sessions)	Left RF	Clinical	Improved motor function and dystonic posture	None	IV	U
Arens et al., 1997 [13]	5–17	Open label (*n* = 15), pure dystonia (*n* = 5), mixed dystonia (*n* = 5)	Cerebral palsy	Ona BTA, 4–6 U/kg	Limbs muscles	Motor function scale (0–4)	Improvement in motor function scale scores	Transient post-injection weakness (N = 2/15, 13%)	IV	U
Heinen et al., 1997 [17]	15–18	Open label (*n* = 6/28)	Cervical dystonia	Abo BTA, 4.8–11.2 U/kg	SC, SCM, TRA	Joint mobility (ROM), Tsui index, global rating scale	Improvement in all outcomes	None	IV	U
Dressler et al., 2001 [22]	20	Case report (*n* = 1)	Mandibular dystonia in HSS	Abo BTA, 400 U	LP, M	Clinical	Improvement in dystonic symptoms and daily activities	None	IV	U
Schwerin et al., 2004 [18]	3–19	Open label (*n* = 28, lower leg dystonia, *n* = 1)	Lower limb dystonia	Rima BTB (large muscles 1000–5000 U, small muscles 250–1000 U)	Not specified	Improved motor function, care, hygiene, orthotic management; correction of cosmetically and functionally distressing limb positions	Goals met in all three categories	Not specified	IV	U
Sanger et al., 2007 [14]	2–15	Open label (*n* = 7)	Cerebral palsy and upper limb dystonia	Rima BTB, 50–200 U/kg	BB, BR	Primary. Maximal velocity of outward reaching Secondary. UDRS (upper limb components), UPDRS (upper limb motor subscale), BFMDS upper limb components, MAS, PQLQ	Increase in maximal velocity of outward reaching Improved BFMDS and UPDRS scores	Transient post-injection weakness (N = 2/7, 29%)	IV	U
Lundy et al., 2009 [15]	2–19	Open label (*n* = 26, 16 with mixed forms)	Cerebral palsy (mixed spasticity and superimposed dystonia)	Abo BTA, 30 U/kg (range, 400–1000 U) Ona BTA, 12 U/kg (range, 100–300 U)	ILIO, AM, medial HAM	PPPQ	Improvement	None	IV	U
Crisci and Esposito 2011 [20]	Not reported	Case report (*n* = 1)	Limb dystonia in PKAN	Abo BTA, 400 U (200 U per muscle)	TP, GM	Clinical	Improvement in motor (hypertonia, internal foot rotation) and social aspects (quality of life)	None	IV	U
Lin et al., 2018 [21]	10	Case report (*n* = 1)	Head and neck dystonia in PKAN	Ona BTA, 180 U (20/30 U per muscle)	SCM, SC, SSC, LS (right) TM, BB, FPL (left)	BADS CP QOL-Child WeeFIM PSI-SF	Improvement in all domains	None	IV	U
Valentine et al., 2020 [16]	8–16	Open label (*n* = 28, 5 with concomitant dystonia)	Cerebral palsy	Ona BTA, 4.5–11 U/kg	Lower limb muscles	GMFCS	Improvement in 21.4% and no change in 78.6%.	None	IV	U
Hull et al., 2021 [19]	1–13	Open label (*n* = 7)	Opisthotonus	Ona BTA, 16.7 to 23.8 U/kg (mean, 19.6)	PM (N = 7/7), SC (N = 5/7) + other muscles	Clinical	Complete resolution of opisthotonus	Transitory neck extensor weakness (14%)	IV	U

Abo BTA: Abotulinum toxin type A. Ona BTA: Onabotulinum toxin type A. Rima BTB: Rimabotulinum toxin type B. AM: adductores magni; BB: biceps brachii muscle; BR: brachioradialis muscle; FPL: flexor pollicis longus muscle; GM: gastrocnemius pars medialis muscle; HAM: hamstrings; ILIO: iliopsoas; LP: lateral pterygoid muscle; LS: levator scapulae muscle; M: mylohyoid muscle; OO: orbicularis oculi muscle; PM: paraspinal muscles; RF: rectus femoris muscle; SC: splenius capitis muscle; SCM: sternocleidomastoideus muscle; SSC: semispinalis capitis muscle; TRA: trapezius muscle; TM: teres major muscle; TP: tibialis posterior muscle; BFMDS: Burke–Fahn–Marsden dystonia scale; MAS: modified Ashworth scale; UDRS: Unified Dystonia Rating Scale; UPDRS: Unified Parkinson’s Disease Rating Scale; PQLQ: Pediatric Quality of Life Questionnaire; ROM: range of motion; PPPQ: Paediatric pain profile questionnaire; GMFCS: Gross Motor Function Classification System; PKAN: pantothenate kinase-associated neurodegeneration; BADS: Barry–Albright dystonia scale; CP QOL-Child: quality of life by primary caregiver proxy-report form of cerebral palsy quality of life for children; WeeFIM: ADL by the functional independence measure for children; PSI-SF: parenting stress of caregivers by parenting stress index short form; HSS: Hallervorden–Spatz syndrome.

## Data Availability

All the data supporting the results are in the figures and tables included in the present manuscript.
